# Blood pressure control and risk of post-stroke dementia among the elderly: A population-based screening study

**DOI:** 10.3389/fneur.2022.956734

**Published:** 2022-08-09

**Authors:** Hao Wu, Zhihong Ren, Jinghuan Gan, Yang Lü, Jianping Niu, Xinling Meng, Pan Cai, Yang Li, Baozhi Gang, Yong You, Yan Lv, Shuai Liu, Xiao-Dan Wang, Yong Ji

**Affiliations:** ^1^Clinical College of Neurology, Neurosurgery and Neurorehabilitation, Tianjin Medical University, Tianjin, China; ^2^Tianjin Key Laboratory of Cerebrovascular and Neurodegenerative Diseases, Department of Neurology, Tianjin Dementia Institute, Tianjin Huanhu Hospital, Tianjin, China; ^3^Department of Neurology, Capital Medical University Electric Teaching Hospital/State Gird Beijing Electric Power Hospital, Beijing, China; ^4^Department of Cognitive Disorder, China National Clinical Research Center for Neurological Diseases, Beijing Tiantan Hospital, Capital Medical University, Beijing, China; ^5^Department of Geriatrics, The First Affiliated Hospital of Chongqing Medical University, Chongqing, China; ^6^Department of Neurology, The Second Affiliated Hospital of Xiamen Medical College, Xiamen, China; ^7^Department of Neurology, Affiliated Traditional Chinese Medicine Hospital of Xinjiang Medical University, Urumqi, China; ^8^Dementia Clinic, Affiliated Hospital of Zunyi Medical University, Zunyi, China; ^9^Department of Neurology, The First Hospital of Shanxi Medical University, Taiyuan, China; ^10^Department of Neurology, The First Affiliated Hospital of Harbin Medical University, Harbin, China; ^11^Department of Neurology, Second Affiliated Hospital of Hainan Medical University, Haikou, China; ^12^Department of Neurology, Hainan General Hospital, Haikou, China

**Keywords:** blood pressure control, post-stroke dementia, hypertension, risk factors, age

## Abstract

**Background:**

Post-stroke dementia (PSD) has adverse effects on the quality of work and life in elderly stroke survivors. There are inconsistent results on the impacts of blood pressure control on the risk of PSD in people aged 65 years and above.

**Objective:**

This study was performed to explore whether poorly-controlled blood pressure was associated with an increasing risk of PSD.

**Methods:**

The study population was enrolled from cross-sectional research conducted in 106 communities of rural northern China. In Phase I, a total of 7,448 people aged ≥65 years, including 830 with stroke history, completed a questionnaire, a physical examination, and a cognitive assessment. Phase II further confirmed the diagnosis of PSD. Well-controlled blood pressure was defined as an average systolic blood pressure of <140 mmHg and average diastolic blood pressure of <90 mmHg over two readings in person. Failure to meet these criteria was considered as poorly-controlled blood pressure.

**Results:**

The crude prevalence rate of PSD among stroke survivors aged 65 years and over was 17.8% [95% confidence interval (CI) 15.2–20.4%]. Among the 830 stroke survivors, the proportions of PSD gradually increased with age and the crude prevalence rates for PSD were 10.2% (95% CI 5.6–14.9%), 14.8% (95% CI 10.1–19.5%), 18.8% (95% CI 14.1–23.5%), and 27.4% (95% CI 20.8–34.1%) in subjects aged 65–69, 70–74, 75–79 and ≥80 years, respectively. Participants in the poorly-controlled blood pressure group were more likely to suffer from PSD (28.4 *vs*.15.3%, *P* < 0.001), be older (75.81 ± 4.97 *vs*. 74.74 ± 5.83, *P* < 0.05), and have a worse cognitive level (22.26 ± 7.05 *vs*. 24.10 ± 6.02, *P* < 0.05). Compared with well-controlled blood pressure patients, poorly-controlled blood pressure in stroke survivors significantly increased risk of PSD (odds ratio = 2.20, 95% CI 1.45–3.32) after adjusting for age, gender, and education.

**Conclusions:**

The crude prevalence of PSD among stroke survivors aged ≥65 years was 17.8% at community level. In addition to lower education level and older age, poorly-controlled blood pressure was also an independent risk factor for PSD among the elderly, which is amenable to intervention. Therefore, it is essential to control blood pressure to reduce PSD incidence.

## Introduction

Stroke and dementia are both common diseases in aging societies (1, 2). People often pay attention to physical disability after stroke (3), while post-stroke dementia (PSD), doubled in the population aged ≥65 years compared to without stroke (4), is often neglected (5). Longitudinal cohorts, based on population- and hospital-based research, showed that PSD incidence increased 1.7–3.0% every year (6). With the aging of society and improved survival from stroke, the numbers of patients with PSD will increase. Although many studies have reported the prevalence of PSD, the results vary from 7% in general population to over 50.0% among hospital patients (6, 7). The sample size, study design (hospital-based or population-based studies), as well as the cognitive assessment time (after stroke 6 months or 1 year) contributed to the inconsistence and great variation. However, there have been a few epidemiological studies on PSD among the elderly at community level in China.

The risk of PSD overlaps with the risk of stroke and dementia. Current studies (8–11) revealed significant predictors of PSD, including age >65 years, low education level, previous cognitive decline, vascular risk factors, stroke features, and neuroimaging factors. Although there have been many studies with a high level of evidence, the influence of some factors on PSD occurrence remains unclear, such as high blood pressure.

High blood pressure, which affects more than 75% of people over the age of 65 years, is the leading risk factor for stroke and many other vascular diseases (12). Thus, high blood pressure likely plays an important role in PSD development, although this association remains unclear (10, 13). In several randomized clinical trials concerning the effects of blood pressure reductions on cognitive outcomes, results have generally been inconclusive (14, 15). Some research suggests that the relationship between blood pressure and dementia may be age dependent (16), with high blood pressure at midlife (age 40–64 years) being associated with an increased risk of late-life dementia (17, 18). However, there is no consensus on the association between blood pressure and dementia in those aged 65 years and above. These varied results may be attributed to differences in subtype and cause of dementia. PSD is mainly the result of ischemia of the brain parenchyma caused by atherosclerotic disease (19). Therefore, our study focuses on the relationship between blood pressure control and PSD among the elderly population, so as to explore the potential modifiable risk factors for PSD.

## Materials and methods

### Participants

This study was conducted in rural Ji County in northern China between April 2019 and January 2020. Using a clustering sampling method, we selected 106 community primary health care centers from 949 villages. All individuals aged ≥65 years in each village (who had been living there for at least 5 years) were selected. Selection was based on the date of birth provided on the residence certificate. Each village had one or two fixed medical staff in the local health centers who were familiar with the health of the villagers. The study was approved by the Committee for Medical Research Ethics at Tianjin Huanhu Hospital and the Tianjin Health Bureau (ID: 2019-40).

### Measures

**Figure 1:**
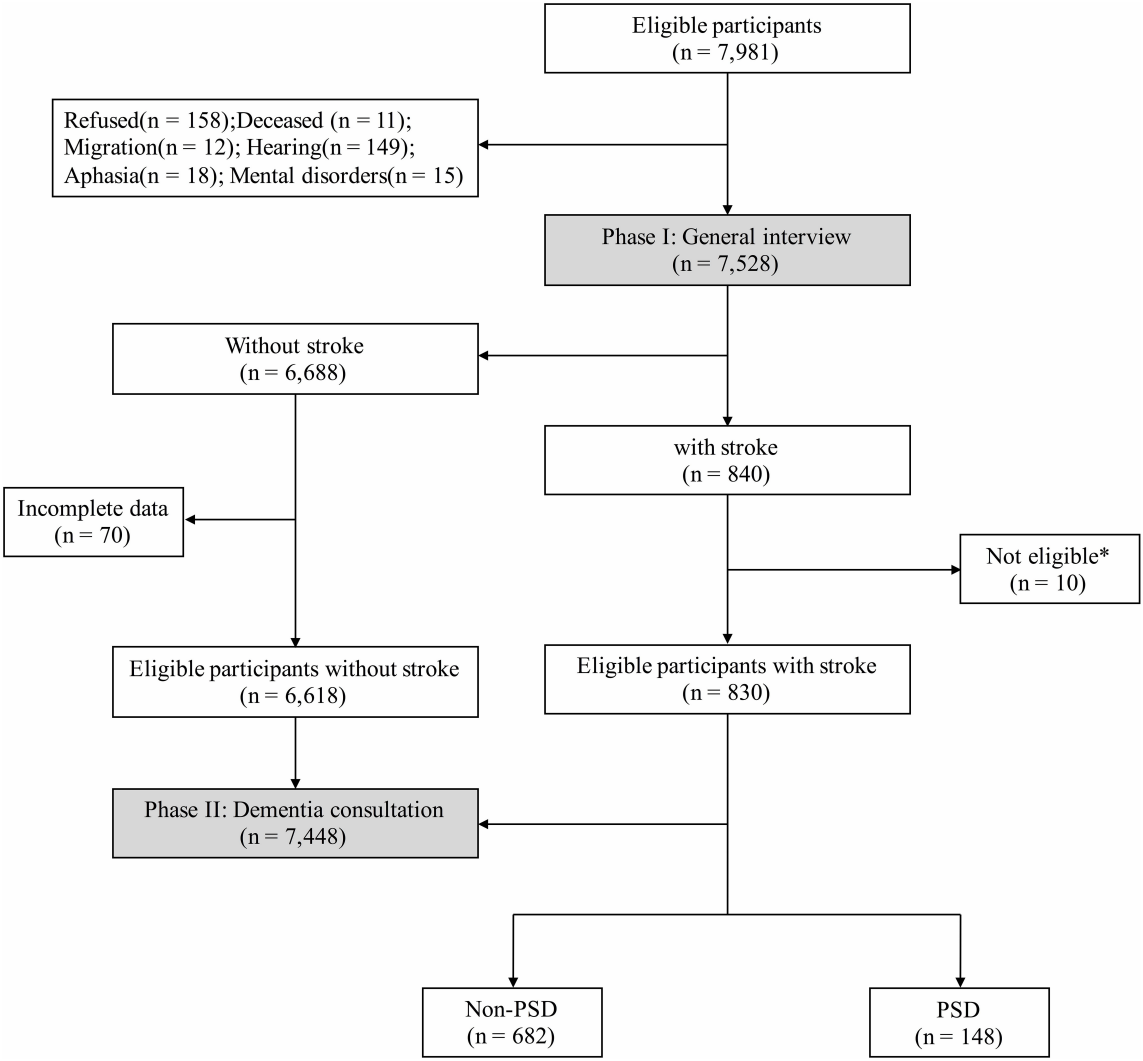
Flowchart of this study. * Not eligible: within 6 months after stroke (*n* = 5); dementia before first-ever stroke (*n* = 2); severe stroke or massive cerebral infarction/hemorrhage (*n* = 3).

There were two phases in the cross-sectional, door-to-door, questionnaire-based survey. All interviewers and experts received the same training on collecting information, neuropsychological assessments, and diagnosis, and participated in a retraining course every 2 months. The total number of participants aged ≥65 years in these communities was 7,891 (Figure 1); however, due to refusal (*n* = 158), death (*n* = 11), migration (*n* = 12), hearing loss (*n* = 149), aphasia (*n* = 18), or mental disorders (*n* = 15), only 7,528 completed the final interview.

#### Phase I: general interview

In Phase I, a centralized medical examination at the health station and an in-person survey were conducted by senior neurologists and medical staff in the local health centers.

During the interview, a questionnaire was completed by all participating patients with the help of their reliable caregivers to obtain information on demographic characteristics, medical history, and habits of smoking and alcohol consumption. Demographic characteristics included name, sex, date of birth, education level, and marital status. Medical history was recorded according to the medical registers, including stroke, hypertension, blood pressure control, diabetes mellitus, heart disease, and dementia. Each participating patient had findings recorded from physical and neurological examinations and had undergone cognitive assessment *via* the Mini-Mental State Examination (MMSE) and Activities of Daily Living (ADL), as well as the Clinical Dementia Rating (CDR) (20). The diagnostic criteria for each disease were consistent with those used in our previous study (21).

Eligible stroke survivors could proceed to Phase II of the study on the same day. The following inclusion criteria were used: (1) age ≥65 years; (2) legally lived in the community for at least 5 years before the study registration; and (3) willingness and ability to participate according to the requirements of the study. Exclusion criteria were (1) known dementia illness history before first-ever stroke; (2) stroke within 6 months; (3) problems affecting cognitive tests, such as severe dysphasia, visual deprivation, hearing loss, and disturbance of consciousness; (4) comorbidities that potentially affect cognitive function, such as malignant neoplasm, serious liver or kidney dysfunction, connective tissue disease, and malnutrition; (5) existing mental disorders; (6) a history of alcohol or drug abuse and poisoning.

#### Phase II: dementia consultation

The second phase was performed by an expert panel. All participants included in this phase were assessed to confirm diagnosis of PSD by obtaining a detailed medical history and performing physical and neurological examinations.

#### Cognitive function assessments

Dementia was diagnosed using the Fifth Edition of Diagnostic and Statistical Manual of Mental Disorders (DSM-V). Assessments were based on data obtained from the interviews, health examinations, previous medical records, and tests for cognitive function and functional capacity. The clinical criteria used for diagnosing PSD included a history of stroke and CT/MRI of brain, and dementia symptoms after stroke occurrence lasting for ≥ 6 months (22).

#### Measurement of incident stroke

Neurologists adjudicated incident strokes by reviewing medical records (inpatient and outpatient medical records) and CT/MRI scans. As part of the routine stroke evaluation for diagnoses in China, patients underwent CT or MRI.

#### Standard for blood pressure control

The survey of blood pressure control was according to the China PEACE Million Persons Project (23). Blood pressure was measured twice on the right upper arm using an electronic blood pressure monitor (Omron HEM-7430; Omron Corporation, Kyoto, Japan), with 1 min between measurements. The mean value was calculated and recorded. And if the difference between the 2 blood pressure readings exceeded 10 mmHg, a third measurement was taken, and the mean value of the last two readings was calculated. Well-controlled blood pressure was defined as an average systolic blood pressure of <140 mmHg and average diastolic blood pressure of <90 mmHg over two readings in person. Failure to meet these criteria was considered as poorly-controlled blood pressure.

### Statistical analysis

Demographics and participant characteristics are presented using descriptive statistics. Demographic variables and risk factors were compared between groups employing *t*-tests, Mann–Whitney tests, or χ2 tests as appropriate. We used multiple logistic regression to analyze the association between blood pressure control and PSD. Statistical significance was defined as *P* < 0.05. All data were analyzed using IBM SPSS Statistics for Windows (Version 22.0; IBM Corp., Armonk, NY, USA).

## Results

### Characteristics of participants

There were 7,891 participants aged ≥65 years old eligible in this study, while a total of 7,448 participants were analyzed after the investigation of Phases I and II (Figure 1), and 830 (11.1%) participants had definite history of stroke with mean age at interview of 74.95 ± 5.69 years. These stroke survivors were more likely to be male (*n* = 395, 47.6%), to have shorter education (5.38 ± 4.34 years), habits of smoking (*n* = 233, 28.1%) and drinking (*n* = 197, 23.7%), and to suffer from diabetes mellitus (*n* = 207, 24.9%), heart disease (*n* = 210, 25.3%), and hypertension (*n* = 596, 71.8%) than those without stroke (Table 1). Additionally, stroke survivors had a poorer cognitive profile, with lower MMSE score (23.74 ± 6.27 *vs*. 25.29 ± 4.90, *P* < 0.001).

**Table 1:** Baseline characteristics and related risk factors of participants in the study.

	**Non-stroke event**	**Stroke survivors**		
		**All stroke survivors**	**Without PSD**	**PSD**
Number of cases, *n* (%)	6,618 (88.9%)	830 (11.1%)	682 (82.2%)	148 (17.8%)
Age at interview, mean ± SD, years	74.40 ± 5.78	74.95 ± 5.69^*^	74.51 ± 5.60	76.97 ± 5.67^‡^
65–69	1,600 (24.2%)	166 (20.0%)	149 (21.8%)	17 (11.5%)
70–74	1,789 (27.0%)	223 (26.9%)	190 (27.9%)	33 (22.3%)
75–79	1,977 (29.9%)	266 (32.0%)	216 (31.7%)	50 (33.8%)
≥80	1,252 (18.9%)	175 (21.1%)	127 (18.6%)	48 (32.4%)
**Gender**, ***n*** **(%)**				
Male	2,761 (41.7%)	395 (47.6%)^*^	332 (48.7%)	63 (42.6%)
Female	3,857 (58.3%)	435 (52.4%)	350 (51.3%)	85 (57.4%)
**Education, mean** **±SD, years**					5.82 ± 4.50	5.38 ± 4.34^*^	5.52 ± 4.33	4.72 ± 4.35^†^
<1	1,260 (19.0%)	174 (21.0%)	130 (19.1%)	44 (29.7%)
1–6	2,770 (41.9%)	376 (45.3%)	321 (47.1%)	55 (37.2%)
≥7	2,588 (39.1%)	280 (33.7%)	231 (33.9%)	49 (33.1%)
**Marital status**, ***n*** **(%)**				
Married	5,120 (77.4%)	627 (75.5%)	514 (75.4%)	113 (76.3%)
Unmarried	1,498 (22.6%)	203 (24.5%)	198 (24.6%)	35 (23.6%)
Smoking, *n* (%)	1,572 (23.8%)	233 (28.1%)^*^	198 (29.0%)	35 (23.6%)
Alcohol consumption, *n* (%)	1,366 (20.6%)	197 (23.7%)^*^	173 (25.4%)	24 (16.2%)^†^
Diabetes mellitus, *n* (%)	926 (14.0%)	207 (24.9%)^**^	171 (25.1%)	36 (24.3%)
Heart disease, *n* (%)	985 (14.9%)	210 (25.3%)^**^	180 (26.4%)	30 (20.3%)
Hypertension, *n* (%)	3,206 (48.4%)	596 (71.8%)^**^	493 (72.3%)	103 (69.6%)
MMSE score, mean ± SD	25.29 ± 4.90	23.74 ± 6.27^**^	25.99 ± 3.41	13.37 ± 6.02^‡^
ADL score, mean ± SD	21.40 ± 5.05	23.40 ± 9.05^**^	20.00 ± 0.00	39.05 ± 12.72^‡^
CDR, mean ± SD	0.21 ± 0.61	0.40 ± 0.86^**^	0.02 ± 0.10	2.14 ± 0.61^‡^

*PSD, post-stroke dementia; SD, standard deviation; MMSE, Mini-Mental State Examination; ADL, activities of daily living; CDR, Clinical Dementia Rating. Significant differences between non-stroke event and all stroke survivor groups:*

* *and*

** *indicate P < 0.05 and P < 0.001, respectively. Significant differences between PSD and without PSD groups:*

† *and*

‡ *indicate P < 0.05 and P < 0.001, respectively*.

According to the PSD diagnostic criteria, 17.8% [95% confidence interval (CI) 15.2–20.4%] of stroke survivors were diagnosed. Participants with PSD were older at interview (76.97 ± 5.67 *vs*. 74.51 ± 5.60 years, *P* < 0.001) than those without PSD. Significantly, participants with PSD had lower educational attainment (4.72 ± 4.35 *vs*. 5.52 ± 4.33 years, *P* < 0.05) and poorer blood pressure control (44.7 *vs*. 23.5%, *P* < 0.001). In addition, the percentage of drinking in PSD was lower than without PSD (16.2 *vs*. 25.4%, *P* < 0.05). There were no differences in gender, marital status, history of smoking, diabetes mellitus, and heart disease between PSD and without PSD groups.

### Blood pressure control and proportions of PSD in different age groups

**Figure 2:**
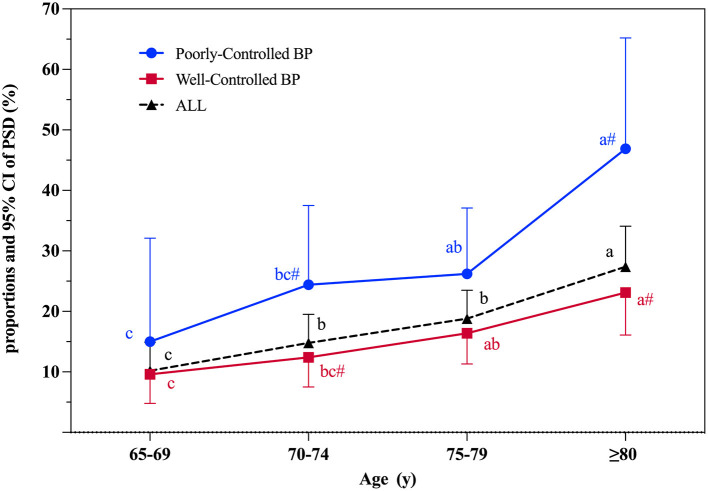
Proportions and 95% CI of PSD according to age at interview. PSD, post-stroke dementia; BP, blood pressure control; 95% CI, 95% confidence interval. Significant differences between well-controlled and poorly-controlled BP groups: # indicates *P* < 0.05. The PSD proportion between different age groups was compared. Used abc letters as the mark letters. If the mark letters are the same, the difference is not significant. If the mark letters are different, the difference is significant (*P* < 0.05).

An age-stratified analysis of the proportions of PSD in different sampled population was conducted (Figure 2). Among the 830 stroke survivors, the proportions of PSD gradually increased with age and the crude prevalence rates for PSD were 10.2% (95% CI 5.6–14.9%), 14.8% (95% CI 10.1–19.5%), 18.8% (95% CI 14.1–23.5%), and 27.4% (95% CI 20.8–34.1%) in subjects aged 65–69, 70–74, 75–79, and ≥80 years, respectively.

The risks of PSD associated with blood pressure control in different age groups are shown in Table 2 and Figure 2. Those in the poorly-controlled blood pressure group were more likely to suffer from dementia (28.4 *vs*.15.3%, *P* < 0.001), be older (75.81 ± 4.97 *vs*. 74.74 ± 5.83 years, *P* < 0.05), and have a worse cognitive level (22.26 ± 7.05 *vs*. 24.10 ± 6.02, *P* < 0.05). Compared with the well-controlled blood pressure group, stroke survivors aged 70–74 years (*P* < 0.05) and ≥80 years (*P* < 0.01) were more likely to suffer from dementia in the poorly-controlled blood pressure group (Figure 2). Significant associations were not observed between blood pressure control and PSD in subjects aged 65–69 or 75–79 years.

**Table 2:** Association of blood pressure control with PSD in stroke survivors.

	**Well-controlled** ** blood pressure** ** (*N* = 668)**	**Poorly-controlled** ** blood pressure** ** (*N* = 162)**	***P-*value**
PSD, *n* (%)	102 (15.3%)	46 (28.4%)	0.000
**Gender**, ***n*** **(%)**							0.071
Male	311 (46.6%)	84 (51.9%)	
Female	357 (53.4%)	78 (48.1%)	
**Age at interview, mean** **±SD, years**					74.74 ± 5.83	75.81 ± 4.97	0.018
65–69	146 (21.9%)	20 (12.3%)	
70–74	178 (26.6%)	45 (27.8%)	
75–79	201 (30.1%)	65 (40.1%)	
≥80	143 (21.4%)	32 (19.8%)	
MMSE score, mean ± SD	24.10 ± 6.02	22.26 ± 7.05	0.003
ADL score, mean ± SD	22.99 ± 8.74	25.07 ± 10.10	0.000
CDR, mean ± SD	0.25 ± 0.82	0.60 ± 0.99	0.001

*PSD, post-stroke dementia; SD, standard deviation; MMSE, Mini-Mental State Examination; ADL, activities of daily living; CDR, Clinical Dementia Rating*.

### Blood pressure control and PSD risk

Alcohol consumption, blood pressure control, age at interview, gender, and education were related to PSD occurrence. The logistic regression models are described in Table 3. We found no significant protective effect on PSD in those with a history of alcohol consumption [odds ratio (OR) = 0.63, 95% CI 0.37–1.08] after adjusting for age, gender, and education. In addition, poorly-controlled blood pressure of stroke survivors significantly increased the risk of PSD (OR = 2.20, 95% CI 1.45–3.32) comparing to those well-controlled, whether adjusted for other risk factors or not.

**Table 3:** Logistic regressions of risks factors for PSD in 830 stroke survivors: OR (95% CI).

**Parameter**	**Model I**	**Model II**	**Model III**	**Model IV**
**Alcohol consumption**				
Yes *vs*. No	0.57 (0.35–0.90)^*^	0.63 (0.37–1.08)		
**Blood pressure control**				
Poor *vs*. Well			2.20 (1.47–3.28)^**^	2.20 (1.45–3.32)^**^
**Age at interview, years**				
65–69		Reference		Reference
70–74		1.59 (0.85–2.98)		1.49 (0.79–2.79)
75–79		2.02 (1.12–3.65)^*^		1.85 (1.02–3.35)^*^
≥ 80		3.21 (1.75–5.90)^**^		3.20 (1.73–5.89)^**^
**Gender**				
Male *vs*. Female		0.98 (0.64–1.51)		1.25 (0.84–1.86)
**Education (years)**				
<1		Reference		Reference
1–6		0.58 (0.36–0.93)^*^		0.59 (0.37–0.95)^*^
≥7		0.73 (0.44–1.20)		0.80 (0.48–1.33)

*OR, odds ratio; 95% CI, 95% confidence interval*.

* *P < 0.05;*

** *P < 0.001. We calculated the associations between Alcohol consumption and PSD in crude model (Model I) and adjusted model after correcting gender, age at interview, and educational years (Model II). Similarly, we also calculated the associations between Blood pressure control and PSD in crude model (Model III) and adjusted model after correcting gender, age at interview, and educational years (Model IV). All data was recorded as OR with 95%CI*.

## Discussion

In this study, we found that the crude prevalence rate of PSD among stroke survivors aged 65 years and over was 17.8% (95% CI 15.2–20.4%) in a rural area of northern China, consistent with previous research results. In addition to low education level and old age, poor control of blood pressure was also an independent risk factor for dementia, which is amenable to intervention. Therefore, PSD prevalence was not low among people over 65 years old at community level, and blood pressure control is essential to reduce PSD occurrence among people aged over 65 years.

Despite many studies on PSD prevalence, there has been a wide range of results. A meta-analysis by Pendlebury and Rothwell (6), excluding pre-stroke dementia, showed that PSD prevalence was 7.4% (4.8–10.0%) in population-based studies of first-ever stroke. Another hospital-based study on stroke recurrence excluded pre-stroke dementia, and PSD prevalence was as high as 41.3% (29.6–53.1%). In China, PSD prevalence was 32.05% according to Montreal Cognitive Assessment and MMSE scales in a population-based study (24). Therefore, it is difficult to accurately estimate PSD prevalence. The reason may be that the analysis methods of these studies differed, including patient characteristics, pre-stroke cognitive level, stroke type, stroke recurrence, diagnostic tools for identifying dementia, and follow-up time. In our research, the crude prevalence rate of PSD was 17.8% (95% CI 15.2–20.4%) in stroke survivors aged 65 years and above, which was lower than the reported PSD rate in China. The possible reason was that we tried to exclude patients with pre-stroke dementia and applied the MMSE scale with slightly lower sensitivity to evaluate cognitive level. In addition, this may be closely related to the time of follow-up for stroke survivors. Previous studies mainly focused on PSD prevalence at 3 or 6 months after stroke. Studies have shown that PSD usually improves within 3–6 months of a stroke, but then worsens over time (25, 26). A comprehensive meta-analysis (27) proposed the term PSD for any dementia that developed following a clinical cerebrovascular event and that the final diagnosis of PSD should be delayed to at least 6 months after the event. Therefore, our study did not include patients at 3–6 months after stroke.

Our results showed that older age and lower educational attainment were risk factors for PSD, but there was no correlation between PSD and gender. This was similar to previous studies. Most studies showed increasing older age was associated with an increased risk of PSD. A systematic review suggested that the risk of incident dementia elevated in relation to older age especially > 80 years [relative risk (RR) = 4.66, 95%CI 2.36–9.22] and age 70–79 years (RR = 2.68, 95%CI 1.52–4.74) vs. age 60–69 years (9). Low education level was an independent predictor of PSD, which was highest in the low education category [hazards ratio (HR) = 1.46, 95%CI 1.18–1.81] followed by intermediate education category (HR = 1.36, 95%CI 1.03–1.81) (28). Although older age and lower education level are recognized risk factors of PSD, it is difficult to change and intervene.

Hypertension is a highly prevalent condition which is an established risk factor for cardiovascular and cerebrovascular disease. In 2020, a meta-analysis (15) included 92,135 participants in 12 trials for analysis of the main results, and found that use of antihypertensive drugs to reduce blood pressure was significantly associated with reducing the risk of dementia or cognitive impairment compared with the control group. Some studies reported that high blood pressure at midlife (age 40–64 years) is associated with an increased risk of late-life dementia (17, 18). Currently, there is no consensus on the association between blood pressure and dementia in those aged 65 years and above (29, 30). On the contrary, some studies (16, 31) suggest that hypotension is associated with greater dementia risk in older age, primarily owing to concerns that aggressive blood pressure reduction could cause hypoperfusion of the brain.

In our study, after adjustments for age, gender, and education, there was an increased PSD risk in those with poorly-controlled blood pressure. Among all stroke survivors, 162 (27.2%) patients had hypertension and poor control of blood pressure, which was lower than previously reported in China (23, 32). However, among the population with PSD, the rate of poorly-controlled blood pressure was 44.7%, and the risk of PSD was 2.20 times that for those with well-controlled blood pressure in the elderly. Moreover, this study found that with increased age, poor blood pressure control was more likely to be associated with dementia, which was significant in the age groups ≥80 and 70–74 years. Although inconsistent with previous results, our study focused on the association of blood pressure control and PSD. The term PSD is defined as dementia that occurs after a stroke, regardless of whether the leading cause is vascular, neurodegenerative, or mixed (33). Early-onset PSD results from a complex interplay between stroke lesion features and brain resilience, whereas delayed-onset PSD is associated mainly with the presence of severe sporadic small-vessel disease and to a lesser extent with Alzheimer's disease pathology or recurrent stroke (34). Therefore, PSD pathogenesis may be more complicated for elderly patients (35). Hypertension causes changes in macro- or micro-vasculature and pathological remodeling, damaging the integrity of vessels and blood brain barrier permeability (13, 36), resulting in hypoxia, oxidative stress, abnormal mitochondrial function, neuroinflammation, and neurodegeneration, which lead to major alterations in the brain microenvironment. Therefore, the cognitive impairment of high blood pressure may be mediated by chronic vascular injury. There seems to be a long-time interval between etiology and clinical results, which may explain the damage of hypertension to cognitive function in the elderly. In addition, a meta-analysis suggested that there was no harmful effect of antihypertensive treatment on cerebral blood flow in older age (37). Secondary analysis of the SPRINT MIND randomized clinical trial study (38) showed that among 547 participants with cerebral blood flow measured at baseline, the average age was 67.5 ± 8.1 years. Compared with standard antihypertensive therapy, intensive antihypertensive therapy was related to increased not decreased cerebral perfusion, which was most obvious among participants with a history of cardiovascular disease. As a result, concerns over cerebral blood flow should not preclude treatment of hypertension in older age.

In addition, the relationship between other vascular risk factors and PSD, such as cholesterol level, blood glucose level, are also worth discussing. But unfortunately, our study was a screening study, no cholesterol level data was collected. Van Vliet et al. (39) have shown that high total serum cholesterol in middle age was related to cognitive impairment and statin therapy was likely to have a benefit on cognitive function via a decrease of cardiovascular pathologies. However, there were no clear consistent relationships between cholesterol and cognitive decline or dementia in this older adult group, nor was there evidence of effect modification by statin use (40). We found that there was non-significant association between diabetes and PSD. Some clinical studies have shown that diabetes was associated with increased dementia risk in stroke, while others reported non-significant relationships (41, 42). Therefore, the level of evidence that controlling glucose level can prevent cognitive impairment is relatively low (22). Therefore, the influence of blood glucose level and cholesterol level on PSD is controversial, and more randomized double-blind trials should be conducted.

## Limitations

There were some limitations in this study. First, although we made the best use of imaging evidence and medical records to identify stroke events with the help of community physicians, some important stroke characteristics (e.g., type, location, and recurrence) were not always available. Second, since we did not perform position emission tomography or cerebrospinal fluid examination on all participants, other types of dementia types such as Alzheimer's disease and dementia with Lewy bodies could not be completely excluded from the included PSD patients. Moreover, MMSE is a short test. Although it is commonly used in cognitive screening, the exact cognitive states according to MMSE score is not accurate enough. If we can follow up further, it will be more meaningful for the diagnosis and prognosis of PSD.

## Conclusion

We found that the crude prevalence of PSD among stroke survivors aged 65 years and over was 17.8% (95% CI, 15.2–20.4%) in a rural area of northern China. In addition to lower education level and older age, poorly-controlled blood pressure was also an independent risk factor for PSD among the elderly, which is amenable to modification. Therefore, it is essential to control blood pressure to reduce PSD incidence. A prospective cohort to explore the relationship between blood pressure control and dementia is necessary in further research, which may contribute to the secondary prevention of dementia.

## Data availability statement

The raw data supporting the conclusions of this article will be made available by the authors, without undue reservation.

## Ethics statement

The study was approved by the Committee for Medical Research Ethics at Tianjin Huanhu Hospital and the Tianjin Health Bureau (ID: 2019-40). The patients/participants provided their written informed consent to participate in this study.

## Author contributions

HW and JG contributed to analysis and interpretation of data and wrote original manuscript. ZR reviewed and revised the manuscript. YLü, JN, XM, PC, YLi, BG, YY, YLv, ZR, SL, and X-DW did the investigation, data management of the center, and revised the manuscript. YJ designed of study, supervised the research process, and revised the manuscript. All authors contributed to the article and approved the submitted version.

## Funding

This work was supported by the National Natural Science Foundation of China [Grant Number 82171182], Science and Technology Project of Tianjin Municipal Health Committee [Grant Numbers ZC20121 and KJ20048], and Tianjin Key Medical Discipline (Specialty) Construction Project (No. TJYXZDXK-052B).

## Conflict of interest

The authors declare that the research was conducted in the absence of any commercial or financial relationships that could be construed as a potential conflict of interest.

## Publisher's note

All claims expressed in this article are solely those of the authors and do not necessarily represent those of their affiliated organizations, or those of the publisher, the editors and the reviewers. Any product that may be evaluated in this article, or claim that may be made by its manufacturer, is not guaranteed or endorsed by the publisher.
