# Corrigendum: Blood pressure control and risk of post-stroke dementia among the elderly: A population-based screening study

**DOI:** 10.3389/fneur.2022.1069667

**Published:** 2022-10-28

**Authors:** Hao Wu, Zhihong Ren, Jinghuan Gan, Yang Lü, Jianping Niu, Xinling Meng, Pan Cai, Yang Li, Baozhi Gang, Yong You, Yan Lv, Shuai Liu, Xiao-Dan Wang, Yong Ji

**Affiliations:** ^1^Clinical College of Neurology, Neurosurgery and Neurorehabilitation, Tianjin Medical University, Tianjin, China; ^2^Tianjin Key Laboratory of Cerebrovascular and Neurodegenerative Diseases, Department of Neurology, Tianjin Dementia Institute, Tianjin Huanhu Hospital, Tianjin, China; ^3^Department of Neurology, Capital Medical University Electric Teaching Hospital/State Gird Beijing Electric Power Hospital, Beijing, China; ^4^Department of Cognitive Disorder, China National Clinical Research Center for Neurological Diseases, Beijing Tiantan Hospital, Capital Medical University, Beijing, China; ^5^Department of Geriatrics, The First Affiliated Hospital of Chongqing Medical University, Chongqing, China; ^6^Department of Neurology, The Second Affiliated Hospital of Xiamen Medical College, Xiamen, China; ^7^Department of Neurology, Affiliated Traditional Chinese Medicine Hospital of Xinjiang Medical University, Urumqi, China; ^8^Dementia Clinic, Affiliated Hospital of Zunyi Medical University, Zunyi, China; ^9^Department of Neurology, The First Hospital of Shanxi Medical University, Taiyuan, China; ^10^Department of Neurology, The First Affiliated Hospital of Harbin Medical University, Harbin, China; ^11^Department of Neurology, Second Affiliated Hospital of Hainan Medical University, Haikou, China; ^12^Department of Neurology, Hainan General Hospital, Haikou, China

**Keywords:** blood pressure control, post-stroke dementia, hypertension, risk factors, age

In the published article, there was an error in affiliation 1. Instead of “Country Clinical College of Neurology, Neurosurgery and Neurorehabilitation, Tianjin Medical University, Tianjin, China,” it should be “Clinical College of Neurology, Neurosurgery and Neurorehabilitation, Tianjin Medical University, Tianjin, China”.

The authors apologize for this error and state that this does not change the scientific conclusions of the article in any way. The original article has been updated.

## Publisher's note

All claims expressed in this article are solely those of the authors and do not necessarily represent those of their affiliated organizations, or those of the publisher, the editors and the reviewers. Any product that may be evaluated in this article, or claim that may be made by its manufacturer, is not guaranteed or endorsed by the publisher.

